# Proton 1H- and Phosphorus 31P-MR spectroscopy (MRS) in asymptomatic HIV-positive patients

**DOI:** 10.7448/IAS.17.4.19577

**Published:** 2014-11-02

**Authors:** Gundolf Schuettfort, Elke Hattingen, Ulrich Pilatus, Christoph Stephan, Timo Wolf, Siri Goepel, Annette Haberl, Stella Blasel, Freidhelm Zanella, Hans-Reinhard Brodt, Markus Bickel

**Affiliations:** 1 University Hospital Frankfurt, Infectious Diseases / ZIM II, Frankfurt am Main, Germany; 2 University Hospital Frankfurt, Neuroradiology, Frankfurt am Main, Germany; 3 Infektiologikum Frankfurt, Infectious Diseases / HIV-Therapy, Frankfurt am Main, Germany

## Abstract

**Introduction:**

HIV infection is accompanied by a variety of neurological disorders. Depression of cell-mediated immunity is followed by the development of central nervous system opportunistic infections/tumours, and frequently by the occurrence of the AIDS dementia complex (ADC). However, the pathophysiology of the emergence of neuro-AIDS is still unknown. Despite the development of cognitive impairments, the early diagnosis, objectification and quantification of the existence and extent of this impairment during infection are difficult to recognize in each individual case. To support the early diagnosis of ADC, there is a need for additional, non-invasive diagnostic methods. In this study, it is of interest to answer the clinically relevant question of whether magnetic resonance spectroscopy can detect changes in the cerebral metabolism of asymptomatic HIV-positive patients and is possibly suitable for the early diagnosis and prevention of HIV encephalopathy.

**Methods:**

A group of 13 asymptomatic, HIV-positive patients with combined antiretroviral therapy (cART) and 13 healthy controls were examined with 2D 1H-MRS and 3D 31P-MRS at 3T. The patients were treated with cART for at least 12 months. Changes in the absolute concentrations of phosphorylated metabolites (ATP), N-acetyl-aspartate, creatine, myo-Isonitol, glutamate/glutamine and choline-containing compounds were compared with that of control subjects.

**Results:**

Asymptomatic HIV-positive patients had significantly lower N-acetyl-aspartate in the white matter in a frontal and parietal target region. The other evaluated metabolites in the 1H MRS showed no significant difference between the HIV-positive patients and healthy controls. The 31P-MRS detected significant elevated values regarding the choline-containing compounds PEth, GPE and PCho.

**Conclusions:**

This spectroscopic study revealed a significantly lower N-acetyl-aspartate in the white matter in a frontal and parietal cerebral target region in asymptomatic, HIV-positive patients as an early sign of neuronal disintegration. The 31P-MRS detected significant elevated values regarding the choline-containing compounds PEth, GPE and PCho as an early sign of gliosis. Furthermore we could show that with the use of 1H-MRS and 31P-MRS cerebral metabolites can be reliably detected and measured in HIV-positive patients. The 1H-MRS and 31P-MRS is therefore suited as a diagnostic tool for early cerebral metabolic changes in HIV-positive patients.

